# Age-dependent effect of high-fructose and high-fat diets on lipid metabolism and lipid accumulation in liver and kidney of rats

**DOI:** 10.1186/1476-511X-12-136

**Published:** 2013-09-18

**Authors:** Uberdan Guilherme Mendes de Castro, Robson Augusto Souza dos Santos, Marcelo Eustáquio Silva, Wanderson Geraldo de Lima, Maria José Campagnole-Santos, Andréia Carvalho Alzamora

**Affiliations:** 1 Departamento de Ciências Biológicas, Instituto de Ciências Exatas e Biológicas, Universidade Federal de Ouro Preto, Morro do Cruzeiro, Ouro Preto, MG 35 400-000, Brazil; 2 Núcleo de Pesquisa em Ciências Biológicas, Universidade Federal de Ouro Preto, Ouro Preto, MG, Brazil; 3 Departamento de Fisiologia e Biofísica, Instituto de Ciências Biológicas, Universidade Federal de Minas Gerais, Belo Horizonte, MG, Brazil; 4 Departamento de Ciências Biológicas, Núcleo de Pesquisa em Ciências Biológicas, Universidade Federal de Ouro Preto, Ouro Preto, MG, Brazil

**Keywords:** High-fat diet, High-fructose diet, Metabolic syndrome, Rats of different ages, NAFLD

## Abstract

**Background:**

The metabolic syndrome (MS) is characterized by variable coexistence of metabolic and pathophysiological alterations which are important risk factors for developing of type II diabetes and/or cardiovascular diseases. Increased of MS patients in worldwide has stimulated the development of experimental models. However, it is still challenging to find an dietetic model that most closely approximates human MS and, in addition, is not yet fully established the effect of different diets of MS in lipid metabolism in rats of different ages. The aim of this study was to evaluate the effect of different diets of MS in lipid metabolism and ectopic fat deposition and define the most appropriate diet for inducing the characteristic disturbances of the human MS in rats of different ages.

**Methods:**

Young (4 weeks old) and adult rats (12 weeks old) were given a high-fat (FAT) or high-fructose diet (FRU) for 13 weeks and biochemical, physiological, histological and biometric parameters were evaluated.

**Results:**

In young rats, the FAT diet induced increased mean blood pressure (MAP) and heart rate (HR), body weight after 6 to 10 weeks, and in the 13th week, increased the liver, mesenteric, retroperitoneal and epididymal fat weights, fasting glucose, alanine aminotransferase (ALT) and aspartate aminotransferase (AST) and reduced HDL cholesterol; and also induced non-alcoholic fatty liver disease (NAFLD) and renal inflammatory infiltrates. In adult rats, the FRU diet induced transient elevations of MAP and HR in the 6th week, and, at 13 weeks, increased fasting glucose, triglycerides, total cholesterol, AST and ALT; increased liver, kidneys and retroperitoneal fat weights; and induced macrovesicular and microvesicular NAFLD, the presence of fat cells in the kidney, glomerular sclerosis, and liver and kidney inflammation. Additionally, the FAT and FRU diets induced, respectively, increases in liver glycogen in adults and young rats.

**Conclusions:**

Our data show that FRU diet in adult rats causes biggest change on metabolism of serum lipids and lipid accumulation in liver and kidney, while the FAT diet in young rats induces elevation of MAP and HR and higher increased visceral lipid stores, constituting the best nutritional interventions to induce MS in rats.

## Background

Metabolic syndrome (MS) is a pathological condition in which three or more of the following risk factors are present: central obesity, high plasma triglyceride levels, low plasma HDL levels, high cholesterol, hyperglycemia, atherosclerosis, non-alcoholic fatty liver disease (NAFLD), endothelial dysfunction, insulin resistance (IR), and/or hypertension [1,2]. These metabolic abnormalities observed during MS are important risk factors for developing cardiovascular disease [3,4] and are associated with the development of type II diabetes [5,6].

The development and establishment of MS are mainly related to the consumption of high-fat diets and/or high-carbohydrate diets [7]. Epidemiological studies have shown that consumption of high-fat diets (≥ 30% of energy from fat) is correlated with high rates of overweight, central obesity and MS [8,9]. Increasing evidence also associates the consumption of a diet high in carbohydrates, such as fructose, with high prevalences of obesity, IR, hypertension and MS [10]. Therefore, the increasing consumption of fructose-rich beverages and/or foods sweetened with table sugar, corn syrup and other preparations and/or processed products containing fructose is of great concern [11].

The increased number of MS patients worldwide has stimulated the development of experimental models that mimic the characteristics of human MS [12], in attempts to understand the biochemical, physiological and pathological alterations involved in the development and maintenance of excess body fat and MS [7]. Several genetic models mimic many of the features of MS occurring in humans, such as obese Zucker rats, obese spontaneously hypertensive rats (Koletsky rats) and Stroke-prone SHR-fatty (fa/fa) rats [13]. However, animal models that develop characteristics of MS without genetic manipulation, but only through consumption of specific nutritionally unbalanced diets are increasingly important [14] for use in simulating the most common cause of human MS.

Despite the large volume of published studies using experimental models of MS, it is still challenging to find a model that most closely approximates human MS. Experimental models of diet-induced MS vary widely in the induction of MS disturbances [15-18], mainly due to the wide variation in the proportion and/or the types of nutrients that compose the diets and/or the different ages of the animals used. This wide variability of the protocols used in studies on MS limits the reproducibility of the dietary treatments used and comparisons of published data, making clear the need for a precise definition of the nutritional intervention to induce in animal models, the disturbances typical of human MS. In addition, is not yet fully established the effect of different diets of MS in lipid metabolism and ectopic fat deposition in rats of different ages.

This study submitted rats of different ages (young and old) to the two main diets used in the literature to induce MS, the high-fat diet [7,19] and high-fructose diet [20,21], and evaluated different biometric, physiological, biochemical and histological parameters in order to define the most appropriate dietary treatment to induce the characteristic disturbances of human MS, and evaluate the effect of different diets of MS in lipid metabolism and ectopic fat deposition in rats of different ages.

## Methods

### Animals

The study used male Fischer rats, newly weaned at 4 weeks of age (young rats, 40 – 60 g) and rats at 12 weeks of age (adult rats, 200 – 250 g) from the Laboratory of Experimental Nutrition (LABNEX) of the Federal University of Ouro Preto (UFOP, Brazil). The animals were kept in individual cages under controlled temperature (25 ± 1°C) and a 12 h – 12 h light–dark cycle in the Animal Science Center (CCA/UFOP). Throughout the experiment, the animals had free access to water and diets. All procedures were performed in accordance with the Guidelines for Ethical Care of Experimental Animals. The protocol was approved by the Animal Ethics Committee of the Federal University of Ouro Preto Protocol No. 2011/31.

### Experimental protocol

Young and adult rats were randomly divided into three groups subjected to different diets: AIN-93 control diet (CT) [22], high-fat diet (FAT) or high-fructose diet (FRU) for 13 weeks. The composition of the diets is shown in Table 1. All groups of adult rats were fed the CT diet from shortly after weaning until the start of the experiment.

The experimental groups were:

1) Young – CT: young rats (4 weeks old) given the control diet (AIN-93G, CT) immediately after weaning during a period of 13 weeks;

2) Young – FAT: young rats (4 weeks old) given a diet containing 40% fat (FAT) immediately after weaning, for 13 weeks;

3) Young – FRU: young rats (4 weeks old) given a diet containing 60% fructose (FRU) after weaning, for 13 weeks;

4) Adult – CT: adult rats (12 weeks old) given the control diet (AIN-93 M, CT) for 13 weeks;

5) Adult – FAT: adult rats (12 weeks old) given a diet containing 40% fat (FAT) for 13 weeks;

6) Adult – FRU: adult rats (12 weeks old) given a 60% fructose diet (FRU) for 13 weeks.

**Table 1:** Diet composition and energy contents of diet

**Ingredients (g/Kg)**	**AIN-93**		**FRU**		**FAT**	
	G (Young)*	M (Adult)*	**Young**	**Adult**	**Young**	**Adult**
Corn starch	529,50	620,70	-	-	-	-
Sucrose	100,00	100,00	-	-	-	-
Fructose	-	-	600,00	600,00	33,0	34,20
Casein	200,00	140,00	200,00	200,00	180,50	180,50
Condensed milk	-	-	-	-	316,00	316,00
Soybean oil	70,00	40,00	40,00	40,00	-	-
Lard	-	-	-	-	370,00	370,00
Fiber (cellulose)	50,00	50,00	-	-	50,00	50,00
Wheat bran	-	-	109,5	108,3	-	-
Mineral mix (AIN-93G-MX)*	35,00	-	35,00	-	35,00	-
Mineral mix (AIN-93 M-MX)*	-	35,00	-	35,00	-	35,00
Vitamin mix (AIN-93G-VX)*	10,00	10,00	10,00	10,00	10,00	10,00
DL-Methionine	3,00	1,80	3,00	1,80	3,00	1,80
Choline Chloride	2,50	2,50	2,50	2,50	2,50	2,50
**Macronutrients (% by weight)**						
Carbohydrate	62,95	72,07	60,00	60,00	20,68	20,80
Fat	5,00	4,00	4,44	4,43	39,53	39,53
Protein	20,00	14,00	21,97	21,95	20,26	20,26
**Macronutrients (% Kcal)**						
Carbohydrate	66,82	75,82	65,25	65,28	15,92	16,00
Fat	11,95	9,46	10,86	10,84	68,48	68,42
Protein	21,23	14,72	23,89	23,88	15,60	15,58
**Kcal/g**	3,77	3,80	3,68	3,68	5,19	5,20
**Kj/g**	15,78	15,91	15,41	15,41	21,73	21,77

*M* maintenance diet, *G* growth diet. * See Reeves [22].

### Arterial pressure measurements

Mean arterial pressure (MAP, mmHg) and heart rate (HR, beats/min) were assessed in awake rats in all groups, in the second, sixth and tenth weeks of the diet, by digital tail plethysmography (Panlab, LE5001). For direct assessment of blood pressure (BP) and HR, rats were anesthetized with a mixture of ketamine and xylazine (50 mg/kg and 10 mg/kg respectively, *ip*) and a polyethylene catheter was inserted into the abdominal aorta through the femoral artery 48 h before the arterial pressure measurement in awake rats. Pulsatile arterial pressure was monitored by a Gould pressure transducer (PM-1000, CWE) coupled to a blood pressure signal amplifier (UIM100A, Powerlab System). The MAP and HR were determined from the arterial pressure wave. All variables were continuously recorded with a PowerLab digital acquisition system (PowerLab 4/25, ADInstruments) with an 800 Hz sampling rate.

### Evaluation of animal and organ weights

All animals were weighed every two weeks until the end of the experimental protocol. At the end of the 13 weeks on the diets, the animals were euthanized, the wet weights of the liver, heart and kidneys (g/100 g rat) were measured, and these organs were then stored for histological analyses. The pancreas and retroperitoneal, epididymal and mesenteric fat depots were dissected and weighed (g/100 g rat).

### Plasma analysis

At the end of the experiment, after euthanasia of animals subjected to an overnight fasting, blood samples (2 to 3 ml) were collected. Then, these samples were centrifuged (8000 *g*, 4°C, 6 min) to separate the plasma for determination of fasting glucose (blood treated with the anticoagulant Glistab, containing EDTA and potassium fluoride) or serum for total and HDL cholesterol, triglycerides, total protein, albumin, creatinine, urea and alanine aminotransferase (ALT) and aspartate aminotransferase (AST). The plasma and serum were aliquoted and stored at (−80°C) to conduct the biochemical analyses. The analyses were performed using individual commercial kits (Labtest, Lagoa Santa, MG, Brazil) according to the instructions provided by the manufacturer.

### Histological analyses

For histopathological analysis, fragments of approximately 1.0 × 1.0 × 0.2 cm of liver, heart and kidneys were fixed in 10% formalin. After 72 h of fixation, the fragments were dehydrated, cleared, and embedded in paraffin. Paraffin blocks were cut into 4 μm thick sections and stained by Hematoxylin and Eosin (H&E) for assessment of architectural damage and the inflammatory process (optical microscopy), or by Periodic Acid Schiff (PAS) for detection of excessive hepatic glycogen. By optical microscopy, the presence or absence of microvesicular and macrovesicular NAFLD, necrosis, inflammation and fibrosis areas in the sections of stained liver tissue were observed. The presence of hepatocytes with glycogen deposits was also evaluated. In the kidneys, the presence or absence of glomerular sclerosis, necrosis, inflammation and fibrosis areas and deposition of lipid cells was evaluated. In the heart, the presence or absence of areas of necrosis, inflammation and fibrosis was evaluated. Representative photomicrographs were obtained with a Leica BM5000 microscope coupled to a Leica DFC 300 FX camera in RGB mode, using a 40× magnification objective.

## Results

### Evaluation of BP and HR

**Figure 1:**
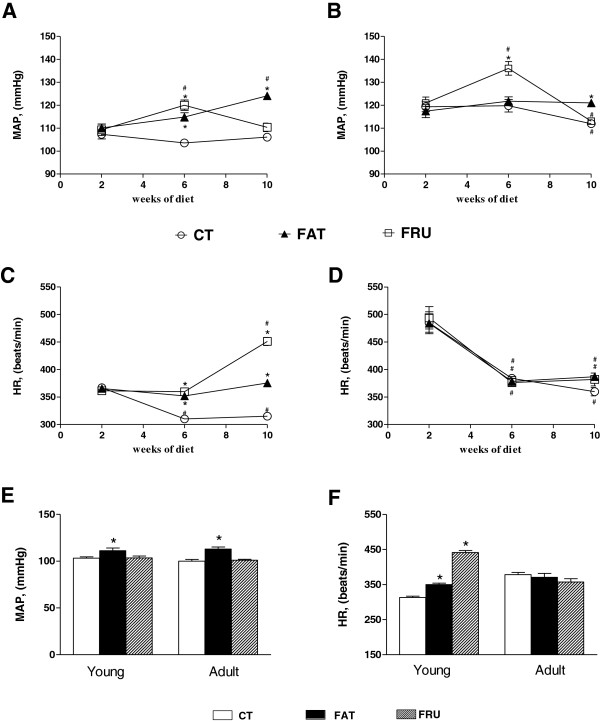
**Evolution of Mean Blood Pressure (MAP) and Heart Rate (HR) evaluated indirectly for tail pletismography (panels A, B, C and D) and directly (panels E and F) in animals that started the experiment at four weeks of age (Young, panels A and C) and at 12 weeks old (Adult, panels B and D) submitted to the control AIN-93 CT (n = 7–10), high-fat (FAT; n = 7–9) or high-fructose (FRU; n = 8–12 adults) diet for 13 weeks.** * P <0.05 compared to the respective group that consumed the CT diet. # P <0.05 compared to data after 2 weeks on the diet.

The indirect evaluation, by tail plethysmography, revealed that both the Young – FAT and the Young – FRU groups showed higher values of MAP and HR in the 6th week of the diet, compared to the Young – CT group. In the tenth week of the diet, the values of MAP and HR of the Young – FAT group remained higher than in the Young – CT, while the Young – FRU group showed only increased HR compared to Young – CT (Figure 1A and C). With respect to adult animals, the Adult – FAT group showed an increase in MAP only up to the tenth week of the diet compared to Adult – CT. However, in the Adult – FRU group the MAP was higher than in the Adult – CT, only in the sixth week of the diet (Figure 1B and D).

The direct evaluation performed at the 13th week of the diet confirmed the results of tail plethysmography and revealed that the Young – FAT animals showed high levels of MAP and HR compared to Young – CT, while the rats of the Young – FRU group showed only an increase in HR. Furthermore, the Adult – FAT rats only showed increased MAP (Figure 1E and F).

### Evaluation of body and organ weights

Adult – CT animals showed increases in the liver weight and retroperitoneal, epididymal and mesenteric fat depots and total visceral fat (TVF) weights compared to the Young – CT group. However, the Adult – CT group showed lower body weight (BW) gain at the end of the 13th week of the diet compared to the Young – CT group (Table 2).

**Table 2:** Relative weights of organs from young and adult rats submitted to different diets

**Weights**	**Young**			**Adult**		
	**CT**	**FAT**	**FRU**	**CT**	**FAT**	**FRU**
Liver	2,46 ± 0,08	2,81 ± 0,07*	2,81 ± 0,11*	3,13 ± 0,09†	3,28 ± 0,72	4,08 ± 0,09*
Kidney	0,27 ± 0,02	0,26 ± 0,01	0,29 ± 0,02*	0,29 ± 0,01	0,32 ± 0,01	0,36 ± 0,01*
Heart	0,30 ± 0,01	0,31 ± 0,01	0,30 ± 0,03	0,27 ± 0,01	0,29 ± 0,01	0,28 ± 0,01
Pancreas	0,78 ± 0,07	0,72 ± 0,01	0,81 ± 0,03	0,84 ± 0,06	0,70 ± 0,06	0,79 ± 0,04
Mesenteric fat	1,05 ± 0,06	1,23 ± 0,05*	0,75 ± 0,10*	1,78 ± 0,18†	1,80 ± 0,14	1,67 ± 0,11
Retroperitoneal fat	1,50 ± 0,06	3,37 ± 0,09*	0,95 ± 0,05*	2,85 ± 0,17†	3,59 ± 0,15*	3,36 ± 0,12*
Epididymal fat	1,63 ± 0,09	2,64 ± 0,06*	1,14 ± 0,07*	2,47 ± 0,17†	2,74 ± 0,16	2,73 ± 0,12
TVF	4,18 ± 0,19	7,24 ± 0,17*	2,85 ± 0,18*	7,09 ± 0,47†	8,13 ± 0,41	7,77 ± 0,24
Weight gain	228,60 ± 16,25	241,90 ± 8,59	74,42 ± 4,83*	163,90 ± 14,09†	132,20 ± 3,80*	162,70 ± 6,06
BW	284,2 ± 15,66	302,1 ± 5,6	131,8 ± 5,48*	402,5 ± 5,99	369,9 ± 6,34	397,6 ± 5,99
N	12	18	12	10	11	10

*N* number of animals, *BW* Body Weight, *TVF* Total visceral fat. * P <0.05 compared to the respective group that consumed the CT diet. † P <0.05 compared to the Young - CT group.

**Figure 2:**
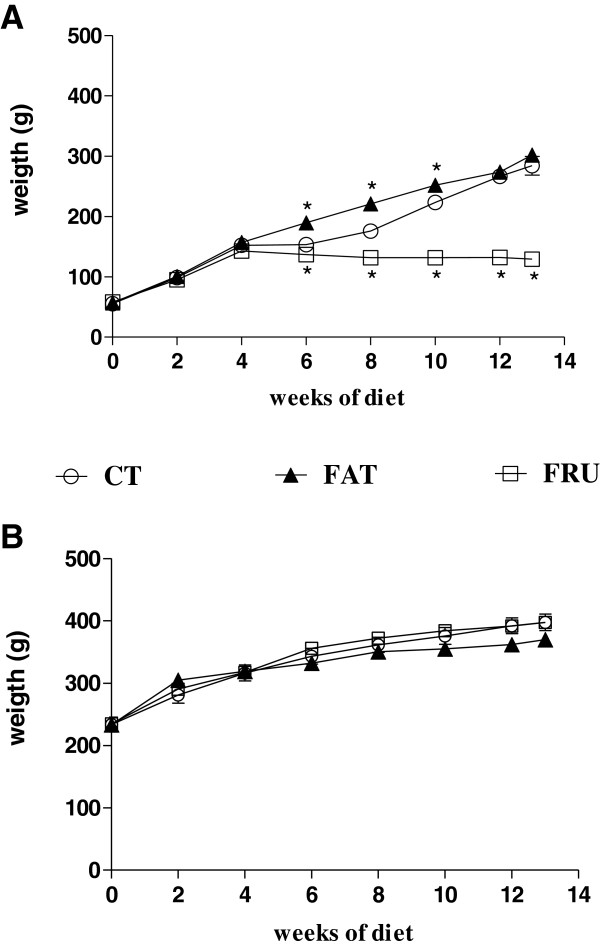
**Evolution of body weight of rats that started the experiment at four weeks of age (Young; panel A) and at 12 weeks of age (Adult, panel B) submitted to the AIN-93 (CT, n = 12), high-fat (FAT, n = 11–18) or high-fructose (FRU, n = 12) diet for 13 weeks.** * P <0.05 compared to the respective group that consumed the CT diet.

The Young – FAT group showed a higher mean BW from the 6th to 10th week of diet (Figure 2), and at the 13th week showed increases in liver weight and mesenteric, retroperitoneal and epididymal fat deposits and TVF weight compared to the Young – CT group (Table 2). The Young – FRU group showed a lower mean BW beginning with the 6th week of the diet (Figure 2), and lower weights of the retroperitoneal, mesenteric, and epididymal fat depots and a decrease in TVF weight and in final BW compared to the Young – CT group. At the 13th week showed increased liver and kidney weights compared to the Young – CT group.

In adult animals, no differences were observed in BW between all groups in any of the weeks of diet evaluated (Figure 2). However, at the 13th week, the Adult – FAT group showed a greater relative weight of the retroperitoneal fat depot and a lower BW gain compared to the Adult – CT group. The Adult – FRU animals showed increases in the weights of the liver, kidneys and retroperitoneal fat depots compared to the Adult – CT group, but no significant differences in the other parameters. In addition, no differences were observed in the relative weights of the heart and pancreas among all groups of young animals (Table 2).

### Plasma analysis

The biochemical analyses performed in the 13th week of diets showed that Adult – CT rats showed high serum triglycerides and albumin compared to the Young – CT group (Table 3).

**Table 3:** Biochemical parameters of young and adult rats submitted to different diets

**Biochemical parameters**	**Young**			**Adult**		
	**CT**	**FAT**	**FRU**	**CT**	**FAT**	**FRU**
Fasting glucose (mmol/L)	5,87 ± 0,14	7,18 ± 0,13*	5,77 ± 0,11	5,84 ± 0,17	5,42 ± 0,63	7,37 ± 0,19*
Triglycerides (mmol/L)	0,39 ± 0,07	0,27 ± 0,03	0,34 ± 0,06	0,64 ± 0,06†	0,68 ± 0,06	1,02 ± 0,11*
Total cholesterol (mmol/L)	2,29 ± 0,24	2,11 ± 0,12	2,26 ± 0,09	1,81 ± 0,16	2,27 ± 0,11	2,97 ± 0,23*
HDL cholesterol (mmol/L)	1,52 ± 0,08	0,37 ± 0,01*	0,38 ± 0,03*	1,63 ± 0,06	1,44 ± 0,24	1,80 ± 0,07
ALT (U/I)	17,31 ± 1,32	24,96 ± 0,92*	30,09 ± 2,78*	18,15 ± 1,80	26,67 ± 3,02*	32,87 ± 1,25*
AST (U/I)	38,56 ± 4,33	72,80 ± 2,27*	87,15 ± 5,35*	30,01 ± 1,01	28,51 ± 2,06	63,43 ± 3,02*
Creatinine (μmol/L)	53,81 ± 5,33	53,44 ± 8,93	57,98 ± 8,13	72,24 ± 14,63	51,06 ± 5,36	52,85 ± 11,85
Urea (mmol/L)	4,33 ± 0,66	4,28 ± 0,56	4,45 ± 0,22	5,05 ± 0,32	5,85 ± 0,50	4,96 ± 0,28
Total protein (g/L)	69,53 ± 1,23	68,17 ± 2,95	45,98 ± 3,03*	67,59 ± 1,04	66,47 ± 1,23	68,19 ± 1,54
Albumin (μmol/L)	352,90 ± 8,27	342,10 ± 20,69	260,0 ± 13,68*	389,00 ± 4,57†	378,60 ± 7,97	373,50 ± 13,63
N	12	18	8	8	10	9

* P <0.05 compared to the respective group that consumed the CT diet. † P <0.05 compared to the Young - CT group.

The Young – FAT animals showed increases in fasting glucose and the ALT and AST transaminases, and reduced serum HDL cholesterol compared to the Young – CT group. The animals of the Young – FRU group also showed decreases in HDL cholesterol and increases in ALT and AST serum, and reduced levels of total protein and albumin compared to the Young – CT group (Table 3).

In relation to adult animals, the Adult – FAT group showed only elevated serum levels of ALT compared to Adult – CT. The Adult – FRU animals showed elevated levels of fasting glucose, triglycerides, total cholesterol, ALT and AST compared to Adult – CT (Table 3).

### Histological analyses

**Figure 3:**
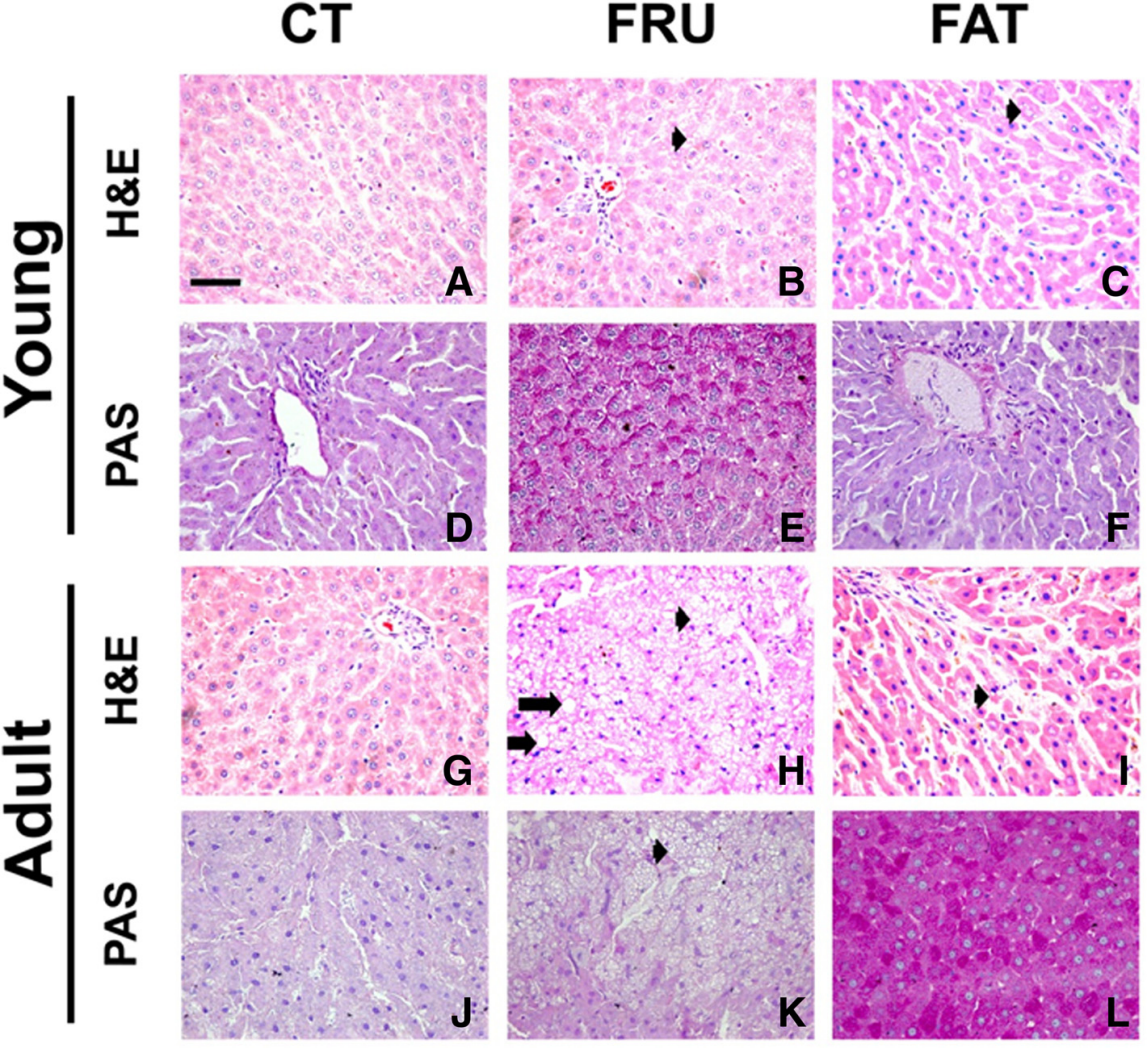
**Histological examination of the liver with hematoxylin and eosin (H & E) staining and determining the excessive deposition of glycogen by periodic acid-Schiff (PAS) in rats that started the experiment at four weeks of age (Young; panels A to F) and at 12 weeks (Adult, panels G to L) submitted to AIN-93 (CT), high-fructose (FRU) or high-fat (FAT) diet for 13 weeks.** The arrowhead () represents the non-alcoholic microvesicular fatty liver disease (NAFLD), and the long arrow () represents the occurrence of macrovesicular NAFLD. Panels **E** and **F** show higher glycogen stores in the Young – FRU and Adult – FAT groups. 40× magnification.

**Figure 4:**
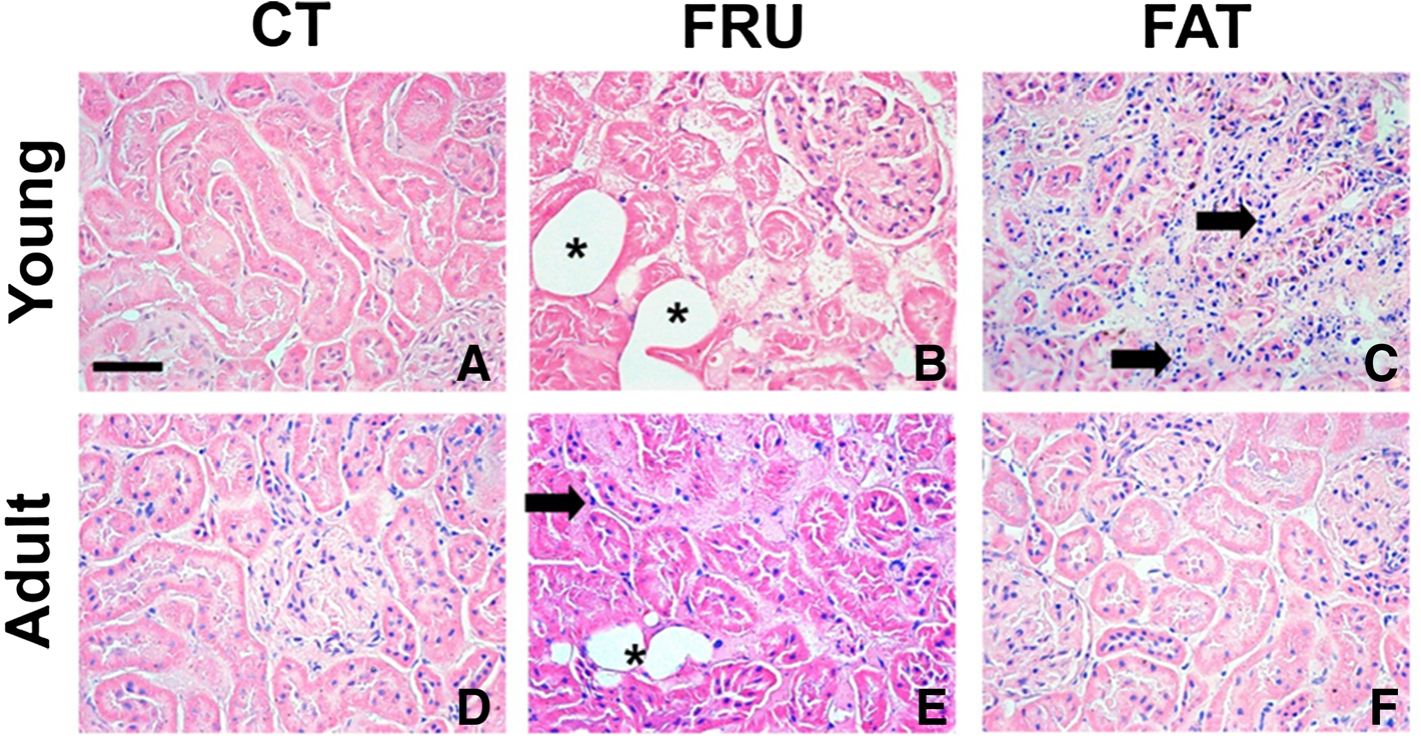
**Histological examination of the kidney with hematoxylin and eosin (H & E) staining in rats that started the experiment at four weeks of age (Young; panels A to C) and 12 weeks of age (Adult, panels D to F) given the AIN-93 (CT), high-fructose (FRU) or high-fat (FAT) diet for 13 weeks.** The arrow () indicates the occurrence of inflammatory infiltration in the Young – FAT and Adult – FRU groups. The asterisk (*) indicates the presence of fat cells in the kidneys of animals in the Young – FRU and Adult – FRU groups. 40× magnification.

The hepatic and renal histology showed that the animals of the Young – FAT group presented microvesicular NAFLD (25%, n = 8), inflammatory infiltrates in the kidneys (50%, n = 8) and hepatic glycogen depots similar to the Young – CT group. The Young – FRU animals showed macrovesicular (20%, n = 10) and microvesicular NAFLD (30%, n = 10), increased hepatic glycogen stores, and showed deposition of fat cells in the kidney (100%, n = 10) (Table 4; Figure 3A to F and Figure 4A to C).

**Table 4:** Percentage of occurence (%) of histological changes in the liver and kidney

	**Liver**					**Kidney**				
	**Tissue inflammation n (%)**	**Tissue fibrosis n (%)**	**Micro-esteatosis n (%)**	**Macro-esteatosis n (%)**	**Excessive glycogen deposition n (%)**	**Adipocytes cell presence n (%)**	**Tissue inflammation n (%)**	**Tissue fibrosis n (%)**	**Glomerular sclerosis n (%)**	**Tissue amyloidosis n (%)**
Young – CT: n=8	*0 (00.0)*	*0 (00.0)*	*0 (00.0)*	*0 (00.0)*	*2 (40.0)*	*0 (00.0)*	*0 (00.0)*	*0 (00.0)*	*0 (00.0)*	*0 (00.0)*
Young – FAT: n=8	*0 (00.0)*	*0 (00.0)*	*2 (25.0)*	*0 (00.0)*	*0 (00.0)*	*0 (00.0)*	*4 (50.0)*	*0 (00.0)*	*1 (12.5)*	*0 (00.0)*
Young – FRU: n=10	*0 (00.0)*	*0 (00.0)*	*3 (30.0)*	*2 (20.0)*	*5 (100.0)*	*10 (100.0)*	*0 (00.0)*	*0 (00.0)*	*0 (00.0)*	*0 (00.0)*
Adult – CT: n=8	*0 (00.0)*	*0 (00.0)*	*0 (00.0)*	*0 (00.0)*	*0 (00.0)*	*0 (00.0)*	*1 (12.5)*	*0 (00.0)*	*0 (00.0)*	*0 (00.0)*
Adult – FAT: n=8	*0 (00.0)*	*0 (00.0)*	*2 (25.0)*	*1 (12.5)*	*5 (100.0)*	*0 (00.0)*	*1 (12.5)*	*0 (00.0)*	*0 (00.0)*	*0 (00.0)*
Adult – FRU: n=10	*3 (30.0)*	*0 (00.0)*	*10 (100.0)*	*6 (60.0)*	*0 (00.0)*	*10 (100.0)*	*5 (50.0)*	*0 (00.0)*	*2 (20.0)*	*0 (00.0)*

Regarding adult rats, the Adult – FAT group presented microvesicular NAFLD (25%, n = 8) and increased hepatic glycogen stores; there were no significant renal histological changes in this group. The Adult – FRU animals showed macrovesicular (60%, n = 10) and microvesicular NAFLD (100%, n = 10), inflammatory infiltrates in the liver (30%, n = 10), and glycogen depots similar to the Adult – CT group. The Adult – FRU group also showed several renal disorders, including the deposition of fat cells in the kidney (100%, n = 10), inflammatory infiltrates (50%, n = 10) and glomerulosclerosis (20%, n = 10), compared to the Adult – CT group (Table 4; Figures 3G, 3L and 4D to 4F). In addition, both the young and adult groups that consumed FAT or FRU diets for 13 weeks showed no significant changes in cardiac histology compared to the control groups (data not shown).

## Discussion

The literature data diverge widely regarding the characterization of MS disorders in rats. These differences are attributable mainly to differences among experimental protocols, such as (1) the variability in the composition of diets; (2) different forms of fructose administration, such as in the diet [23] or water [24]; (3) duration of dietary treatments, from weeks [24] to months [15]; (4) use of different rodent models [15,23]; and different age of animals, i.e., young [17,23] or adults [24]. To minimize these biases, in the present study we compared several biochemical, physiological, histological and biometric parameters of newly weaned young rats and adult rats submitted to the FRU and FAT diets, i.e., those that are most often used to induce disturbances related to MS. Taken together, our data revealed that FRU diet for adult rats and FAT diet for young rats are the best nutritional interventions to induce MS in rats and differently change the lipid metabolism and the ectopic fat deposition in the liver and kidney in rats of different ages.

Studies in animals and humans have shown that overweight and central obesity, characteristic of MS, are associated with important changes in autonomic regulation, such as an increase in the sympathetic nervous system activity [25] and a decrease in the parasympathetic nervous system activity [26]. In addition, consumption of a high-fat diet increases the MAP and HR in both adult obesity-prone rats (32% fat diet) [27] and in humans [28]. Similarly, our results showed that the Young – FAT group increased MAP and HR from the sixth week of the diet.

Published work has shown that a high-fructose diet induced an increase in systolic BP [29,30] and MAP in rats [31,32]; however, D’Angelo et al. [33] showed that adult Sprague–Dawley rats given a diet containing 66% fructose for 8 weeks did not show increases in BP and HR. In the present study, the FRU diet induced a transient increase in MAP only in the sixth week of the diet, in both young and adult animals. This suggests that counter-regulatory mechanisms to increased BP are efficiently activated, so that from the tenth week on, the groups that consumed the FRU diet exhibited BP values similar to their respective control groups.

Interestingly, in this study in the second week was observed in all control animals, especially in the adult group, increased in HR. The probably explanation must be the tail plethysmography methodology used for evaluating cardiovascular parameters. This methodology could be a source of greater stress for adult animals in relation to young animals. Furthermore, the second measurement (6 weeks) onwards, the level of HR in control animals was decreased, probably because the animals have adapted to the procedure, for this reason was carried also direct evaluation of MAP and HR in the 13th week of the diets.

In addition, previous studies have shown that young C57B1/6J mice fed a diet rich in saturated fats, for 7 weeks, developed central obesity especially [34]. Similarly, our data showed that the Young – FAT animals showed increases in BW only in the 6th, 8th and 10th week of the diet, and increased fat depots (mesenteric, retroperitoneal and epididymal) in the 13th week. Our data, together with the literature reports, suggest that the FAT diet induces considerable central obesity, which seems to be related to the deleterious factors associated with MS, rather than to the increase in BW per se [14].

Previous studies showed that adult rats consuming a high-fructose diet (60 g/100 g) for 8 to 10 weeks showed increases in BW [35,36]. However, Moura et al. [21] found that adult rats fed a high-fructose diet (60%) for 8 weeks increased the amount of retroperitoneal fat, but not BW. Similarly, our study showed that Adult – FRU rats did not have increased BW, but did show an increased retroperitoneal fat depot.

The high-fat diet reduces the expression of insulin receptors, inhibits the oxidation of fatty acids (FA) in skeletal muscles, diminishes the mRNA expression and intracellular protein content of GLUT4, and reduces the translocation of GLUT4 to the cell membrane [37,38], factors that may be responsible for the observed hyperglycemia and hyperinsulinemia in animals [39]. In our study, the Young – FAT animals showed elevated plasma levels of fasting glucose, suggesting, together with the evidence from the literature, that the FAT diet alters glucose metabolism, mainly in young animals. Moreover, the FAT diet was not effective in increasing serum triglyceride (TG) in both young and adult rats. Similar findings [40,41] showed that a high-fat diet can also reduce the synthesis of VLDL in hepatocytes, since excess TG consumed can be directed to an oxidative route to form ketones and carbon dioxide. Another plausible explanation is that the high-fat diet has provided low amount of carbohydrates for synthesis of triglycerides and/or the high concentration of saturated fat in the FAT diet could somehow reduce the hepatic secretion of VLDLs [41].

A high-fructose diet greatly increases the amount of this monosaccharide in the liver, independently of insulin action. This event accelerates the production of pyruvate and glycerol 3-P, which in turn stimulates *de novo* lipogenesis, leading to up-regulating of lipogenic enzymes that increase the synthesis, esterification, secretion and accumulation of cholesterol and FA in hepatocytes [42]. Our data agreed with these findings, and showed that the Adult – FRU group had increased serum levels of TG and cholesterol.

The Young – FRU rats had low levels of total protein and albumin, and showed no change in fasting glucose, total cholesterol and TG. These data, combined with the reduction in visceral fat depots and BW in these animals, suggest that the FRU diet is not the most appropriate for induction of MS in young rats. This concords with the findings of Moura et al. [21], who noted that a high-fructose diet induces MS more effectively in adult than in young rats.

NAFLD induces increased hepatic transaminases, nonalcoholic steatohepatitis (NASH), hepatic inflammation, and fibrosis, in both humans and animals [43]. In addition, Lieber et al. [44] demonstrated the presence of NAFLD and NASH in rats that consumed a high-fat diet (71% of energy from fat) for three weeks. Similarly, our data showed that the Young – FAT group had increased liver weight and showed microvesicular NAFLD along with high serum levels of ALT and AST. These data showed that young rats subjected to a FAT diet, in addition to presenting important changes characteristic of MS, such as central obesity and hyperglycemia, also shows damage and ectopic fat deposition in the liver.

Previous studies showed that the FAT diet decreases insulin sensitivity, while the high ratio of saturated fatty acid induces hepatic steatosis [45,46] and hepatic IR [47]. Studies in dogs have found that a high-fat, high-fructose diet impairs glucose uptake, lactate production, the flow of the glycolytic pathway, and the synthesis of hepatic glycogen [47], through interfering with the action of insulin in the liver [45]. Similarly, our findings show that although the rats in the Young – FAT group showed elevated fasting glucose, and consumed high levels of simple carbohydrates in the FAT diet, this group showed no increased glycogen stores, unlike the Adult – FAT group, which showed normal fasting glucose and high glycogen depots. This suggests that the young animals showed greater alterations in the hepatic metabolism in comparison to the adult rats.

The Adult – FRU animals showed more types of liver abnormalities, including increased liver weight, higher percentages of occurrence of and microvesicular and macrovesicular NAFLD, and the presence of inflammatory infiltrates, along with increases in ALT and AST serum levels. Similarly, previous studies in rats consuming diets with high fructose concentrations, both in the feed (60%) [48] and in drinking water (10%) [49], showed microvesicular NAFLD [43,50], intralobular inflammation, and increased expression of IL-6 and TNF-α in the liver [43]. These results together confirm that the consumption of high-fructose diets induces NAFLD and NASH; and in addition, our data showed that these disturbances were most evident in adult rats.

In the early stages of MS induced by fructose, the synthesis of P-trioses increases, which exceeds the oxidative capacity of the liver, leading to stimulation of glucose (50%), lactate (25%) and TG production and glycogen synthesis (15%) [50,51]. Accordingly, Francini et al. [52] showed that in Wistar rats given drinking water containing 10% fructose over three weeks, glycogen stores in the liver increased. However, in more advanced stages, the large input of fructose in the liver produces an allosteric negative regulation of the enzyme phosphofructokinase, reducing the uptake of glucose by the liver [53], increasing hepatic gluconeogenesis, and inducing a high intrahepatic accumulation of lipids, which, in turn, occupy a large volume within hepatocytes and disorganize the hepatic structure and functions such as glycogen storage, as well as reducing hepatic IGF-1 synthesis and inducing hepatic IR [50,54]. Our data showing an increase in glycogen stores of the Young – FRU group, unlike the results for the Adult – FRU group, suggest that these groups are at different stages of MS and hepatic IR induced by fructose. Adult animals appear to be more susceptible to the development of alterations in the hepatic metabolism of glucose and lipids.

Several studies [55-57] have shown that an increase in BP and development of central obesity are followed by renal disorders, such as vasodilation, glomerular hyperfiltration and inflammation. Similarly, our data showed that although serum levels of creatinine and urea did not change, the Young – FAT group showed renal inflammatory infiltrates, along with central obesity and increases in BP and HR, suggesting that there is an association between these factors.

Evidence shows that adult rats consuming a high-fructose diet (60 g/100 g) [24] and diabetic rats at different ages [58] also have various renal impairments, such as hypertrophy, arteriolopathy, cortical vasoconstriction, and hypertension and glomerular hyperfiltration [59] due to increased blood glucose. Our findings that Adult – FRU rats had renal alterations such as accumulation of fat cells, increases in kidney weight, glomerular sclerosis and inflammatory infiltrates, along with elevated blood-glucose levels, reinforce the idea that glycosylation of proteins, the increased release of proinflammatory cytokines, oxidative stress, and the accumulation of lipid peroxidation products may be the cause of kidney damage [58,60]. Regarding the accumulation of fat cells in the kidneys on the groups that consumed the FRU diet, still is not fully understood the exact mechanism by which the FRU diet induced it, but we believe that some renal glucose transporters, such as GLUT2, GLUT5, NaGLT1, SGLT4 [61,62] and the recently reported SGLT5, which is exclusively expressed in the kidney and transports fructose and mannose [63], may be involved [64].

In the present study, we observed no significant changes in cardiac histology in any group submitted to the FAT or FRU diets. Similarly, studies by Carroll et al. [65] and Mellor et al. [66], which submitted, respectively, adult rats to a high-fat diet (32% kcal fat) and to a diet with 60% fructose, also found no hypertrophy [66] or changes in heart function [65].

## Conclusions

Taken together, our results showed that FRU diet in adult rats causes biggest change in metabolism of serum lipids and induces ectopic fat accumulation in liver and kidney, while the FAT diet in young rats induces elevation of MAP and HR and increased visceral lipid stores, constituting the most effective nutritional interventions in inducing, to a large extent, the biochemical, physiological and histological features of human MS.

Statistical analysis was performed using Prism 5 for Windows (GraphPad Software, Inc., San Diego, CA, USA). Data are expressed as means with their standard errors of the mean for animals given each diet. All parameters were analyzed by one-way ANOVA. Post-test comparisons were made using a Bonferroni multiple-comparison test. A *P* value < 0.05 was considered statistically significant.

## Competing interests

The authors declare that they have no conflict of interest.

## Authors’ contributions

UGMC participated in developing protocol, data collection, data analysis and interpretation of data and writing manuscript. ACA contributed in conception, co-ordination, supervision and design of the study, acquisition of funding and critically revising the manuscript. WGL carried out for histological analysis, MES carried out for biochemistry analysis, RASS and MJCS contributed in acquisition of funding and critically revising the manuscript. All authors read and approved the final manuscript.
